# What happens after hepatitis C eradication?

**DOI:** 10.1186/1742-4690-9-S1-I19

**Published:** 2012-05-25

**Authors:** Stanislas Pol

**Affiliations:** 1 Hepatology Unit, Cochin Hospital, Paris, France

**Keywords:** Hepatitis C virus /Pegylated Interferon /Ribavirin/Protease Inhibitors

## 

The treatment of hepatitis C virus infection (HCV) by a combination of pegylated interferon and ribavirin, according to early viral kinetics, leads to a sustained virological response (SVR) in more than 50% of patients with chronic infection. This SVR is a complete recovery of the infection but more than 50% of genotype 1-infected patients do not achieve SVR.

A better understanding of the viral cycle, and the characterization of viral enzymes which are potential targets, resulted in the development of new molecules, direct acting antiviral drugs (DAA) targeted against HCV, either specific of genotype 1 (NS3/NS4A protease inhibitors and NS5B polymerase inhibitors) or with a wider spectrum (NS5A or entry inhibitors), and non-specific antivirals (new interferons, cyclophilin inhibitors). The results of these phase II and III trials which clearly demonstrated a 20 to 30% increase in the SVR rate of genotype 1-infected patients, either naive or treatment experienced.

These new drugs has now been approved by the end of 2011, after a temporary approval for compassionate use in cirrhotic genotype 1 patients with previous relapse or partial response to the combination therapy and a new turn appears with the “interferon free regimens” which combine different direct acting antivirals. The complete virologic recovery which is achieved with all the antiviral treatments clearly reduces the rates of liver-related morbidity and mortality but, in the absence of biopsy-proven cirrhosis reversal, the risk of occurrence of hepatocellular carcinoma is still present and requires a regular US follow-up in those patients with extensive fibrosis or cirrhosis for an early screening. In the experienced patients with active replication, new antivirals are mandatory to reduce the risks of complications, including liver transplantation, which is high, especially in patients with rapid fibrosis.

In the future, the main limitations of triple therapy will be safety (cutaneous rash or anemia which may be controlled), cost, compliance, viral resistance, and drug-drug interactions that must be avoided by educating patients and physicians.

